# Impacted Mandibular First Molar: A Rare Riddle

**DOI:** 10.7759/cureus.31680

**Published:** 2022-11-19

**Authors:** Parmarth M Sonpal, Bhushan P Mundada, Nitin D Bhola, Ranjit Kamble, Jeni Mathew

**Affiliations:** 1 Oral and Maxillofacial Surgery, Sharad Pawar Dental College and Hospital, Datta Meghe Institute of Medical Sciences, Wardha, IND; 2 Orthodontics, Sharad Pawar Dental College and Hospital, Datta Meghe Institute of Medical Sciences, Wardha, IND

**Keywords:** piezosurgery, orthodontic tooth movement, mandibular first molar, cone beam computed tomography, impaction

## Abstract

Tooth impaction incidence is in the range of 5.6 to 18.8% of the population. Eruption failure of the first permanent molar is very rare; the prevalence is 0.01% of the population. The permanent molars are of particular importance in providing adequate occlusal support as well as coordinating facial growth. Failure of the eruption of permanent molars might lead to an array of complications like a reduction in the vertical dimension, extrusion of the antagonist teeth, a posterior open bite, inclination and resorption of adjacent teeth, and cyst formation. Various treatment modalities for impacted teeth include periodic observation, orthodontic relocation, and partial dislocation. More invasively, surgical exposure and extraction of teeth before prosthetic treatment may be performed. It is imperative to diagnose and manage the condition early, as delayed treatment may result in a myriad of problems, like a decreased force of the spontaneous eruption, a decreased percentage of treatment success, and a prolonged period of treatment, increasing the complications furthermore. Because of the importance of permanent molars, eruptive guidance is required before impacted tooth extraction. This article summarizes a case in which the surgical-orthodontic combined approach to the impacted mandibular first molar avoided the need for prolonged orthodontic treatment that would have required repositioning the deeply impacted first molar to the dental arch. As an outcome, patient satisfaction improves.

## Introduction

The phenomenon wherein a tooth that is entirely or partly unerupted is positioned against another tooth, bone, or soft tissue such that its further eruption is unlikely, as described by its anatomical position, is known as impaction [1]. Permanent tooth impaction is not very uncommon. The impaction of third molars is the most common, followed by maxillary canines, mandibular premolars, mandibular canines, maxillary premolars, maxillary central incisors, and mandibular second molars [1].

The incidence of tooth impaction varies from 5.6 to 18.8% of the population. Failure of eruption of the first permanent molar is rare; the prevalence is 0.01% in the case of the first permanent molar [2]. However, the impaction of the first mandibular molar (0.01% prevalence) and the first and second maxillary molars (0.02% prevalence) is quite rare [3]. The permanent molars are particularly important for providing sufficient occlusal support and coordinating facial growth [2]. Impactions from such teeth can lead to a variety of complications. Early diagnosis and treatment are essential because delayed treatment will invite various problems like decreased spontaneous eruptive force, longer treatment duration, and increased complications. Due to the importance of permanent molars, eruptive guidance before the extraction of impacted teeth is necessary [4].

Excessive removal of bone may be necessary for extracting deeply impacted molars, resulting in complications like inferior alveolar nerve injury and iatrogenic mandibular fractures. A modified surgical approach may be warranted in such a challenging scenario [5].

## Case presentation

**Figure 1:**
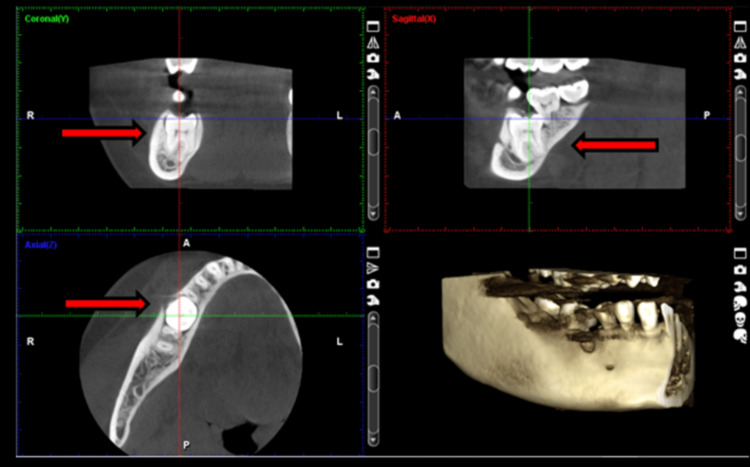
Cone beam computed tomography (CBCT) image of an impacted mandibular first molar

A 21-year-old female patient, systemically healthy, was referred to the department of oral surgery for an impacted first molar extraction. The condition was incidentally identified radiologically prior to orthodontic treatment (Figure 1).

**Figure 2:**
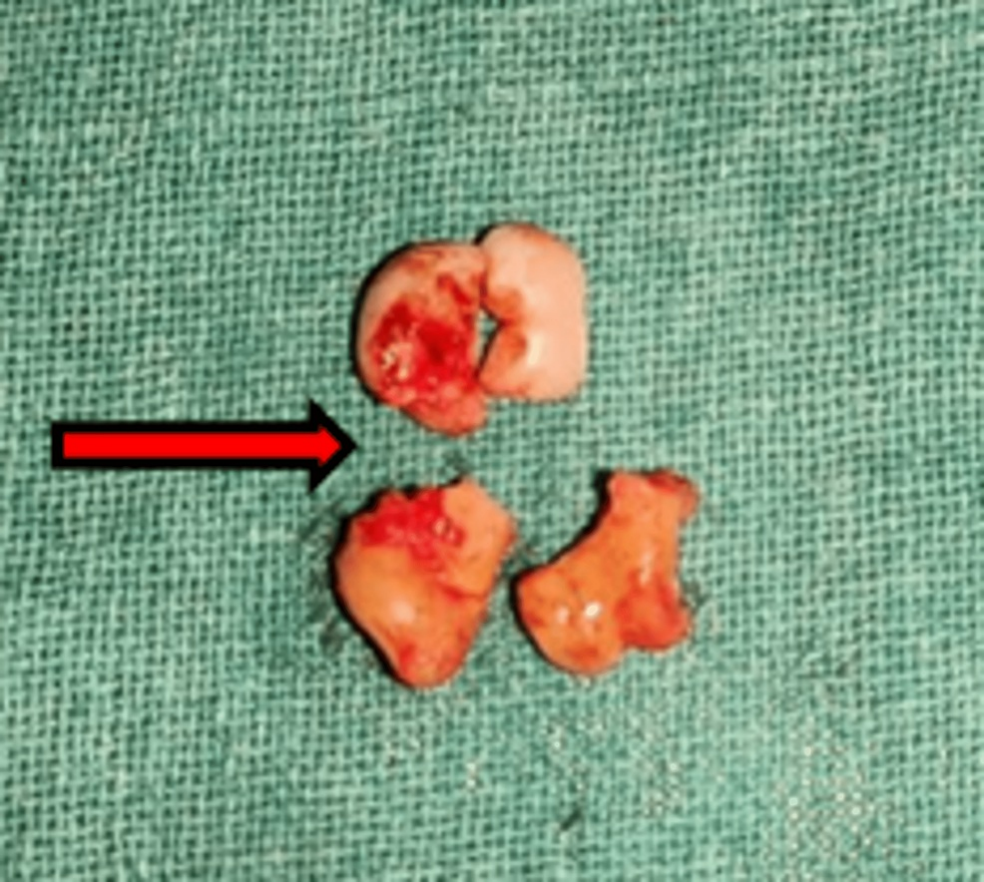
Extraction of the impacted mandibular first molar in sections

After a thorough intraoral as well as radiologic examination, a decision was made that the affected tooth should be extracted with a conventional technique. The biggest challenge in the case was preserving maximum bone for orthodontic movement while minimizing damage to an adjacent tooth as less space was available, all the while avoiding nerve injury. All the risks were explained to the patient, and consent was signed. Under local anesthesia, a crevicular incision was made from the distal aspect of the right mandibular first premolar to the distal part of the right mandibular second molar, and one reliving incision was made mesial to the first premolar, making it an envelope flap design. The full-thickness mucoperiosteal flap was raised, and the mental nerve was identified and preserved. Irrigation was done with normal saline to reduce the heat and remove the debris. Buccal and occlusal bone guttering and shaving were done to expose the tooth up to the cement-enamel junction. Odontectomy was started with a micromotor and round burr number eight under copious irrigation with normal saline. To reduce the patient's cost, a micromotor was used for tooth sectioning. First, the crown and root were separated by a horizontal cut, and then the crown was sectioned into two parts by a vertical cut and delivered buccally without harming the adjacent tooth. Subsequently, the root was split into two parts and delivered coronally and buccally (Figure 2).

**Figure 3:**
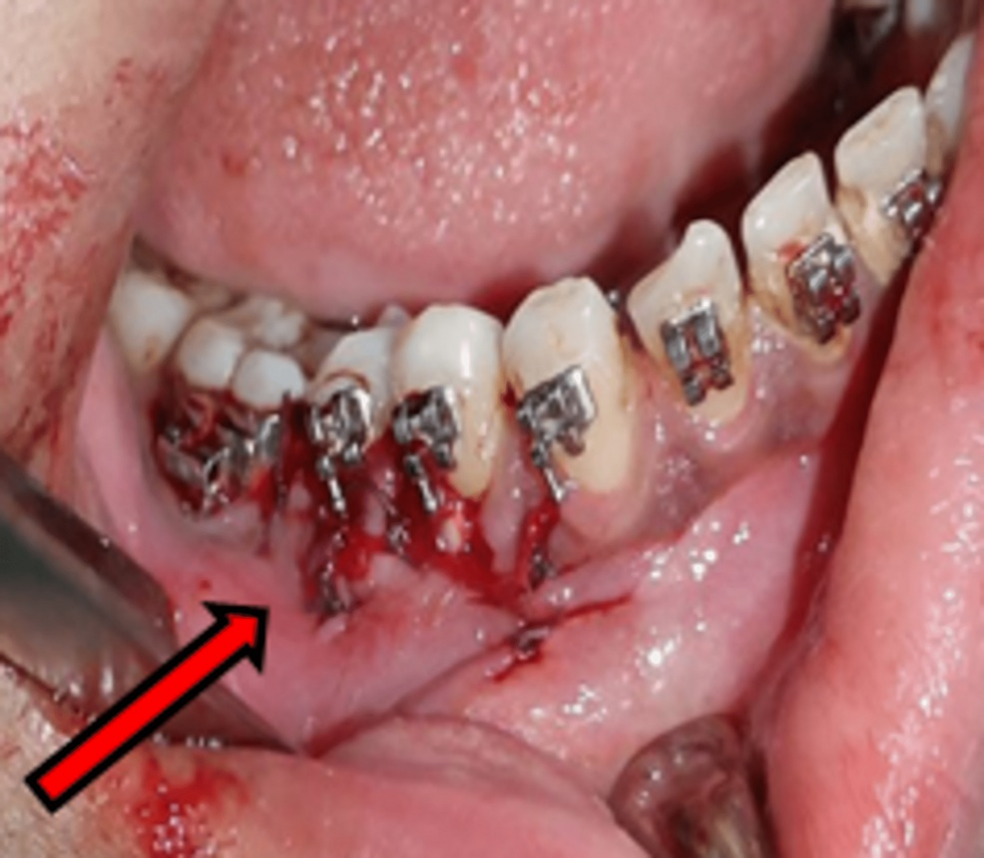
Closure of a previously raised mucoperiosteal flap

After inspecting the socket for debris and bone pieces, thorough toileting of the socket was done using normal saline and betadine solution. The previously raised flap was closed primarily with 3-0 vicryl sutures (Figure 3).

**Figure 4:**
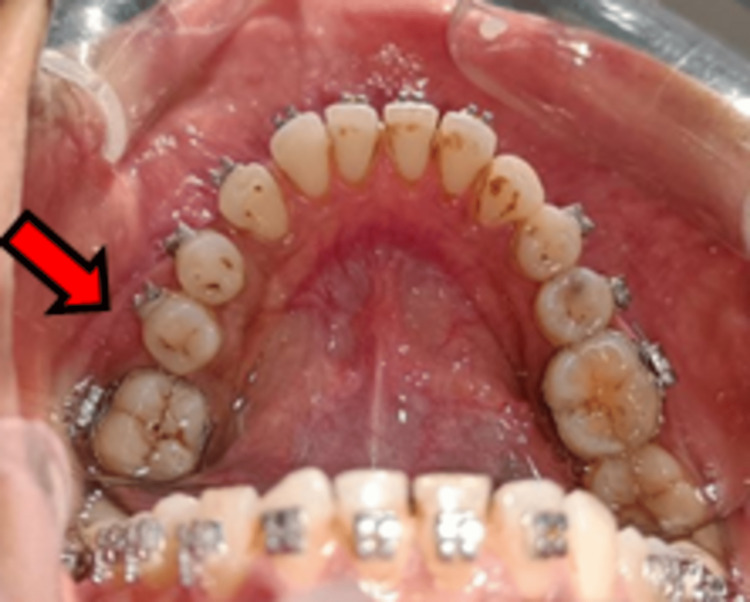
Complete mandibular arch at six months follow-up.

To relieve pain after surgery, a combination of aceclofenac (100 mg), paracetamol (325 mg), and serratiopeptidase (15 mg) was administered twice daily. With the exception of temporary paraesthesia of the inferior alveolar nerve, which was confirmed via a two-point discrimination test, recovery was nearly uneventful. Tablets of vitamin B complex with B12 twice a day were advised for 30 days. The paraesthesia was resolved in six months (Figure 4).

## Discussion

Impaction results from a physical obstruction and a shortage of space, which might cause the second and third molar follicles to collide. Impaction may also occur if there is excess space between the first and second molars as they grow, most likely because the first molar's distal root is necessary for the appropriate eruption of the second molar's crown. Physical, clinical, and radiological examinations should be used to rule out all likely reasons if any mandibular molars do not erupt at the expected time or if others have already done so.

Ectopic location, obstructions in the eruption path, and failures in the eruption process are the three main factors that Andreasen et al. have identified for eruption disturbances [3]. For diagnostic purposes, Raghoebar et al. divided tooth eruption disturbances into the following three groups: impaction, primary retention, and secondary retention [6]. A variety of local and systemic factors are related to tooth eruption failure. One of the etiologic factors is heredity [3]. Recently, some familial cases of primary failure of the eruption have been shown to have mutations in the parathyroid hormone receptor 1 [3]. Malocclusion, problems with deciduous dentition, the positioning of adjacent teeth, a lack of space in the dental arch, idiopathic factors, supernumerary teeth, odontomas, or cysts are a few local causes of eruptive failure. Unfortunately, it was difficult to establish a firm differential diagnosis for these atypical eruption patterns either clinically or radiographically before starting treatment. The first molar impaction is caused mainly by an ectopic eruption, while the second molar impaction is caused primarily by an arch length deficiency [6].

The term "primary failure of eruption" has been used to describe an uncommon disorder that has been linked to unerupted teeth, most frequently molars. It is described by Profitt and Vig as a condition where the eruption mechanism is unable to function normally, causing non-ankylosed teeth to fail to erupt. Infraocclusion, a significant posterior open bite, non-syndromic eruption failure without mechanical obstruction, and an inability to move the impacted teeth orthodontically are some of the characteristics. Because they are difficult to make, most diagnoses are made by exclusion [7].

The surgeon faces difficulties while trying to remove an impacted mandibular first molar without adequate preoperative preparation. The difficulty of extracting an impacted tooth depends on its depth. Deeply impacted molars can be removed surgically using a variety of methods, including the extraoral approach, lingual split, and buccal osteotomy. In all instances, the conventional removal of impacted teeth causes lingual nerve paresthesia in 0.9%-11% of cases and inferior alveolar nerve (IAN) paresthesia in 1.6%-3.3% of cases. [8]. The extraoral technique puts the facial nerve's marginal mandibular branch in danger and could result in nerve injury.

## Conclusions

The surgical management of these rare conditions has always been difficult and demands a step-by-step repair. For this case, we took a combined surgical-orthodontic approach. Our goal was to cause minimal damage to adjacent teeth while preserving as much bone as possible for orthodontic tooth movement. Before beginning the intervention, a proper sequence of surgical procedures for the desired outcomes must be established. As a result, it is clear that no specific treatment algorithm can be developed for the defects presented in the current case. Each case is unique and requires a tailored treatment plan.
